# Validation of self reported diagnosis of hypertension in a cohort of university graduates in Spain

**DOI:** 10.1186/1471-2458-5-94

**Published:** 2005-09-12

**Authors:** Alvaro Alonso, Juan José Beunza, Miguel Delgado-Rodríguez, Miguel Angel Martínez-González

**Affiliations:** 1 Department of Preventive Medicine and Public Health, University of Navarra, Pamplona, Spain; 2 Department of Epidemiology, Harvard School of Public Health, Boston, MA, USA; 3 Service of Internal Medicine, University Clinic, University of Navarra, Pamplona, Spain; 4 Department of Health Sciences, University of Jaén, Jaén, Spain

## Abstract

**Background:**

The search for risk factors of hypertension requires the study of large populations. Sometimes, the only feasible way of studying these populations is to rely on self-reported data of the outcome. The objective of this study was to evaluate validity of self-reported diagnosis of hypertension in a cohort of university graduates in Spain.

**Methods:**

The Seguimiento Universidad de Navarra (SUN) Study is a cohort of more than 15,000 university graduates in Spain. We selected a random sample of 79 cohort participants who reported a diagnosis of hypertension and 48 participants who did not report such diagnosis (76% participation proportion). Then, we compared information on the self-reported diagnosis of hypertension and hypertension status as assessed through two personal blood pressure measurements and an interview. Additionally, we compared self-reported and measured blood pressure levels with intraclass correlation coefficients and the survival-agreement plot.

**Results:**

From those 79 reporting a diagnosis of hypertension, 65 (82.3%, 95% CI 72.8–92.8) were confirmed through conventional measurement of blood pressure and the interview. From those 48 that did not report a diagnosis of hypertension, 41 (85.4%, 95% CI 72.4–89.1) were confirmed as non hypertensives. Results were similar among men and women, but were worse for overweight and obese individuals, and for those with a family history of hypertension. The agreement between self-reported and measured blood pressure levels (as a continuous variable), as estimated by the intraclass correlation coefficient, was 0.35 for both systolic and diastolic blood pressure.

**Conclusion:**

Self-reported hypertension among highly educated participants in a cohort study is a relatively valid tool to assess the hypertensive status of participants. However, the investigators should be cautious when using self-reported blood pressure values.

## Background

High blood pressure or hypertension (HT) is a major health problem in our environment.[1] The low degree of awareness among the population and the difficulties to comply with prescribed treatments, stress the importance of primary prevention of this disease.[2]

The search for risk factors of incident HT requires the study of large populations. Sometimes, the only feasible way of studying these populations is to rely on self-reported data of the outcome. As a consequence, it is of the utmost importance to assess the validity of that information.

The validity of the self-reported diagnosis of HT has been assessed in several populations, including a subsample of the EPIC-Spain cohort.[3] Results vary depending on the population and on the gold standard used (conventional measurement of blood pressure (BP) or examination of medical records).

Our objective was to assess the validity of self-reported diagnosis of HT in a random sample of the participants in the Seguimiento Universidad de Navarra (SUN, University of Navarra Follow-up) study, a cohort study in Spain.

## Methods

### The SUN Study

The SUN Study is a dynamic cohort of university graduates, recruited and followed up through mailed questionnaires. The main objective of the study was to assess the association between a Mediterranean dietary pattern and the risk of cardiovascular disease, diabetes, obesity, and HT. Its methods have been extensively described elsewhere.[4] Briefly, beginning in December 1999, all graduates of the University of Navarra, Registered Nurses in Navarra, and members of other professional associations received a questionnaire and a letter of invitation, explaining the objectives and design of the study. At December 2004, 17,500 had answered the initial questionnaire, and the recruitment is permanently open. Every other year, a follow-up questionnaire is mailed to each participant, gathering information about new medical diagnosis and changes in exposures of interest. The SUN Study was approved by the Institutional Review Board of the University of Navarra, and conforms to the principles embodied in the Declaration of Helsinki.

### Questionnaires

The baseline questionnaire gathered information about sociodemographic variables, anthropometric measures (weight, height), lifestyle factors (smoking, physical activity), diet, and clinical variables. The participants were asked whether they had ever received a medical diagnosis of HT, their habitual use of medications, and their most recent BP measurement (choosing among the following categories, in mm Hg: lower than 100, 101–110, 111–120, 121–130, 131–140, 141–150, 151–160, 161–175, greater than 175 for systolic BP; lower than 60, 61 to 70, 71 to 80, 81 to 90, 91 to 100, 101 to 110, 111 to 120, 121 to 130, greater than 130 for diastolic BP). This question did not differentiate between casual BP determinations or more formal BP measurements carried out according to diagnostic protocols.

The follow-up questionnaire inquired about new diagnosis of HT asking whether the participant had been diagnosed by a physician since the last questionnaire.

### Validation study

In September 2003, there were 2,929 SUN participants living in the metropolitan area of Pamplona (postal codes beginning by 310). Among them, 151 referred to be hypertensive (5.2%). Based in results from the literature, [3,5,6] we assumed that 80% of them would be true cases of HT. In order to obtain an 8% precision in the estimates and expecting 10% of non-response, we selected a random sample of 107 individuals that referred a diagnosis of HT in the baseline or in the two-year follow-up questionnaires, residing in the metropolitan area of Pamplona, that were alive and did not participate in a previous study on validation of diet and physical activity information. Similarly, assuming that 90% of those not reporting HT would be true normotensives, an 8% precision in the estimate and a non-response rate of 10%, we randomly selected 61 individuals, with the same inclusion/exclusion criteria than for the self-reported hypertensives.

We sent them a letter with the objectives of the validation study, an informed consent form and a contact information form (e-mail address, telephone number and hours to be called), together with a postage-paid envelope. After three months, non-respondents were sent a second or third mailing if needed. Finally, we tried to contact non-respondents by phone or email. A hundred and fifty two (response rate 90.5%) individuals accepted to participate in the validation study, 97 (response rate 90.7%) among the hypertensives and 55 among normotensives (response rate 90.2%).

After the participants gave their written consent, an appointment was made at their home, working place, or the Check up Unit at the University Clinic for the BP measurement. A structured interview was done including two BP measurements and a questionnaire about medication use and lifestyle issues related to HT. Two medical doctors (AA, JJB) carried out the field work, including the BP measurements, from September 2003 to November 2004. At the time of the measurement, both study physicians were unaware of the hypertensive status of the participant as defined in the questionnaire.

During the first minutes of the interview and with both the participant and the investigator sited down, the investigator explained the participant the objectives of the study, the type of interview, the BP measurements procedure and the confidentiality of the information. Then, the first BP measurement was done using an automatic BP measurement device Omrom M4-I. This device has been previously validated. [7] After another five minutes, used to complete the rest of the questionnaire, the second BP measurement was done. Hypertensive patients under drug therapy were not asked to stop using antihypertensive medication, because current use of antihypertensive medication was considered confirmatory of being true hypertensives.

A total of 127 participants (83.6%) completed the validation protocol. The remaining 25 (7 normotensives and 18 hypertensives) were lost either because they changed their contact information and could not be located, they refused to participate, failed to make an appointment with the investigator or had changed the place of residency out of the region and could not make an appointment in Pamplona. Final participation among hypertensives and normotensives was, respectively, 78.7% and 73.8%.

### Definition of self-reported HT and 'true' HT

We considered a participant had self-reported HT when s/he answered to have been diagnosed as hypertensive by a physician either in the basal or in the follow-up questionnaire. Otherwise, s/he was considered as non-hypertensive.

We considered a participant as true hypertensive when the average of both BP measurements was ≥140 mmHg for systolic BP and/or ≥ 90 mmHg for diastolic BP, when s/he was currently using antihypertensive drug treatment or when s/he presented a medical report with a diagnosis of HT.[8]

### Statistical analysis

We computed the proportion of confirmed cases of HT as the number of those who reported a diagnosis of HT and had HT according to our gold standard, divided by all those reporting a diagnosis of HT. Similarly, we computed the proportion of confirmed non hypertensives as the number of those who did not report a diagnosis of HT and were non hypertensives according to our gold standard, divided by the total number of individuals non reporting a HT diagnosis. We studied agreement between self-reported and measured BP using a random-effects model intraclass correlation coefficient [9] and the survival-agreement plot proposed by Luiz et al.[10] In the survival-agreement plot, the absolute difference X_i _between BP measures was plotted in the x-axis against the proportion of pair of observations with an absolute difference equal or lower than X_i _using the Kaplan-Meier method.[10] We also used the modification proposed by Llorca and Delgado to detect bias in any of the measurement methods.[11] According to the proposed modification, we separated those observations with self-reported BP higher than measured BP, and those with measured BP higher than self-reported BP. Then, we compared absolute differences both groups using the log-rank test.

To compute the sensitivity and the specificity of the self-reported diagnosis of HT, we estimated the expected distribution of true and false positives and negatives in the sampled population, based on the sampling fractions and the observed percentages of confirmed diagnosis. Then, we computed the kappa coefficient and the true prevalence of HT in that population. Confidence interval (CI) for the prevalence of HT was estimated as suggested by Cochran for stratified sampling.[12]

## Results

We included 70 men and 57 women in our analyses. Mean age was 53 among those self-reporting HT and 37 among those not reporting a HT diagnosis (range 22–83 and 23–72 respectively). A total of 60 (47.3%) had a BMI ≥ 25 kg/m^2^. Table 1 shows the main characteristics of the study participants.

**Table 1:** Characteristics of participants in the validation study by self-reported HT status.

	Self-reported HT (n = 79)	Non self-reported HT (n = 48)
Women (%)	36.8	54.2
Age		
≤ 40 y (%)	16.5	68.8
41–55 y (%)	30.4	22.9
>55 y (%)	53.2	8.2

Body mass index		
<25 kg/m^2 ^(%)	40.5	72.9
≥ 25 kg/m^2 ^(%)	59.5	27.1

Family history of HT (% yes)	45.6	27.1

We confirmed 65 (82.3%) of the 79 self-reported HT cases (95% CI 72.8–92.8%). Among 48 participants who did not report a HT diagnosis in the questionnaires, 41 (85.4%, 95% CI 72.4–89.1%) could be considered normotensives according to our gold standard (table 2). In this last group, when the cut-off point for HT was 160/95 instead of 140/90, the proportion of confirmed normotensives increased to 97.9% (95% CI 88.7% to 100%).

**Table 2:** Hypertension status and validity of self-reported hypertension according to relevant variables

	N (%)	% Confirmed HT (95% CI)§	P-value*	% Confirmed non-HT (95% CI)†	P-value*
Total	127 (100)	82.3 (72.8–92.8)		85.4 (72.4–89.1)	

Men	70 (55.1)	85.4 (72.8–92.8)	0.53	77.3 (56.6–89.9)	0.22
Women	57 (44.9)	78.6 (60.5–89.8)		92.3 (75.9–97.9)	

Age			0.27		0.73
≤ 40	46 (36.2)	69.2 (42.4–87.3)		87.9 (72.7–95.2)	
41–55	35 (27.6)	79.2 (59.5–90.8)		81.8 (52.3–94.9)	
>55	46 (36.2)	88.1 (75.0–94.8)		75.0 (30.1–95.4)	

Body mass index (kg/m^2^)			0.84		0.37
<25	67 (52.7)	81.3 (64.9–71.1)		88.6 (74.0–95.5)	
≥ 25	60 (47.3)	83.0 (70.9–91.1)		76.9 (49.7–91.8)	

Family history of HT			0.01		0.004
No	78 (61.4%)	72.1 (57.3–83.3)		94.3 (81.4–98.4)	
Yes	49 (38.6%)	94.4 (81.9–98.5)		61.5 (35.5–82.3)	

Biomedical degree			0.72		0.32
No	101 (61.4%)	81.0 (69.6–88.8)		81.6 (66.6–90.8)	
Yes	26 (38.6%)	87.5 (64.0–96.5)		100.0 (72.2–100.0)	

HT: hypertension. CI: confidence interval.

§True positives (according to both methods)/Positives (according to self-reporting)

† True negatives (according to both methods)/Negatives (according to self-reporting)

* Pearson's chi squared

There were no antihypertensive drug users among those reporting no hypertension, and 46% of those reporting a diagnosis of hypertension (36 out of 79) were taking antihypertensive drugs at the time of the interview. Among the remaining 43, only 14 (33%) had their BP measurements under 140/90 and were not receiving drug treatment for hypertension.

The proportion of confirmed hypertensives was higher among those groups with an expected higher prevalence of HT (men, older people, and those with high BMI or with a family history of HT). The proportion of confirmed normotensives followed an inverse pattern (table 2).

Taking into account our sampling fractions and assuming our estimate for the proportion of confirmed hypertensives, we expected that 124 out of the 151 individuals reporting a medical diagnosis of HT in the source population were true hypertensives (true positives) and 27 were normotensives (false positives).

Likewise, the number of true normotensives (true negatives) in the source population would be 2373 (from 2778 self-reported normotensives) and 405 would be hypertensives (false negatives). Based on these assumptions, the values for sensitivity, specificity and kappa coefficient would be 0.23, 0.99, and 0.31, respectively. The prevalence of HT in this population was 18.1%.

In spite of the categorization used to collect self-reported data about BP (see above), we calculated the intraclass correlation coefficient and its 95% CI to assess the absolute agreement between self-reported BP and directly measured BP as a continuous variable (Table 3). In general, the correlation between self-reported and directly observed information was low, similar for systolic and diastolic BP and higher for men than women.

**Table 3:** Intraclass correlation coefficients (95% CI) between self-reported Blood Pressure* and directly measured blood pressure §

	Systolic BP	Diastolic BP
Total	0.35 (0.09–0.55)	0.35 (0.16–0.51)
Men	0.36 (0.01–0.62)	0.45 (0.20–0.64)
Women	0.30 (0.01–0.54)	0.24 (-0.07–0.51)

≤ 55 years	0.29 (0.02–0.52)	0.23 (-0.04–0.47)
>55 years	0.27 (-0.03–0.53)	0.41 (0.12–0.63)

BP: blood pressure. CI: confidence interval.

* Seven categories for systolic BP (from <100 to >150 mmHg) and 7 categories for diastolic BP (<60 to >130) were offered for participants' choice in the questionnaire. We assigned to each category its middle point (95 to 155 mmHg for systolic and 55 to 135 mmHg for diastolic BP) in order to compute the correlation coefficients.

§ The average of two measurements taken 5 minutes apart.

**Figure 1:**
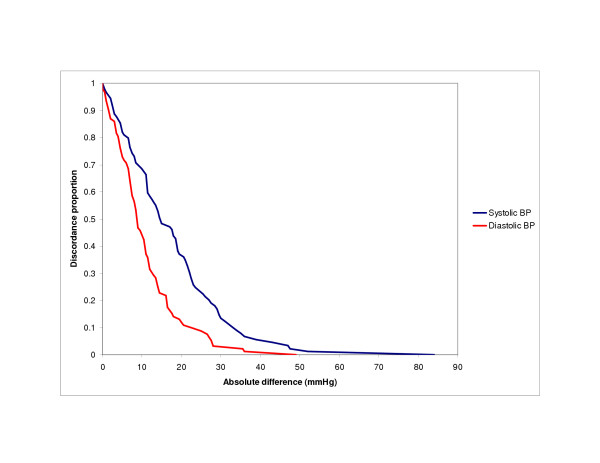
Survival-agreement plot, as proposed by Luiz et al.[10] The x-axis shows the absolute difference between self-reported and measured blood pressure (BP), and the y-axis shows the proportion of observations with differences that are at least the observed difference. Separate lines for systolic and diastolic BP.

Finally, we used the survival-agreement plot to depict graphically the agreement between self-reported and BP measurements (Figure 1). Using the modification of this method proposed by Llorca and Delgado to detect bias, we noted that measured systolic BP tended to be higher than self-reported systolic BP (log-rank test, p = 0.0005). However, this bias was not apparent for diastolic BP (log-rank test, p = 1.00).

## Discussion

Our findings showed an acceptable degree of confirmation of self-reported diagnoses of HT, but the overall agreement was not very high. Particularly, our assumptions for HT prevalence and taking into account the sampling fractions, the sensitivity and the kappa coefficient were low. On the other side, specificity was excellent. Although these results may seem discouraging, we should consider that the use of self-reported diagnoses with low sensitivity but very high specificity in a cohort study do not represent a substantial drawback, because it is very likely that HT, as other chronic diseases, will end eventually showing themselves up during the follow-up of participants. Then, in this particular setting, it would be more important to retain a high specificity, hoping that in the long-term new cases of HT will eventually be diagnosed. In addition, it is important not to forget that all except one of our false negative cases disappeared when the cut-off point for HT was 160/95 instead of 140/90 mmHg, and that there were no individuals taking antihypertensive medication among those reporting normal blood pressure.

Several studies with different methodology have evaluated the validity of self-reported diagnosis of HT. For example, in the EPIC-Murcia cohort, the kappa coefficient between self-reported and medical record-based diagnosis of HT was 0.58, but the investigators did not personally measured the BP of participants, as we did, because their gold standard were only the clinical records.[3] In the South Carolina Cardiovascular Disease Prevention Project, the sensitivity, specificity and positive and negative predictive values were, respectively, 79, 91, 76 and 93 for white women, and 62, 91, 75, and 85 for white men, with no differences between overweight and normal weight subjects.[13] In a sample of Finnish individuals, self-reported HT was confirmed reviewing medical records, obtaining similar results.[6] In the National Health and Nutritional Examination Survey III, the sensitivity for the self-reported diagnosis of HT was 71% and the specificity 90%.[14] Other studies have found similar results. [15-18] Finally, in the Nurses' Health Study and the Health Professional Follow-up Study, with a design similar to the SUN Study, the observed concordance rates among true HT diagnosis and self-reported cases of HT were comparable to ours. [5,19]

Our study has several drawbacks. First, the number of study subjects was relatively small and, thus, validity estimations had wide confidence intervals. Particularly, the separate analysis for different subgroups should be interpreted cautiously. Second, our 'gold standard', two isolated BP measurements, has a limited validity. Actually, HT diagnosis should be based on multiple BP measurements, taken on separate occasions. [8,20] Third, our study design did not allow the direct computation of confidence intervals for sensitivity, specificity and the kappa statistic. On the other side, the high educational level of our study participants ensures that health care utilization and, consequently, HT diagnosis are not influenced by educational status. And, finally, the physicians that performed the BP measurements were unaware of the questionnaire answers, making both assessments of HT diagnosis completely independent, a condition required for validation studies.

The observed agreement between observed and self-reported values of systolic and diastolic BP, as expressed by the intraclass correlation coefficient and the survival-agreement plot, was not high. However, BP levels have a high within-person variability and, in fact, BP is very difficult to track in a population (tracking being defined as the stability of a certain variable over time or the predictability of later values from earlier measurements).[21] In fact, systolic BP measurements tended to be higher than self-reported BP in our population, probably due to a real increase in BP levels over time and also due to a possible white-coat effect.[22]

Finally, we acknowledge that some misclassification will always exist in the self-reported diagnosis of HT. But, on the other side, the study of large populations would be unfeasible if we could only rely on conventional measurements, given the high amount of resources required to perform an accurate diagnosis of HT. The trade-off between precision and sample size has to be kept in mind.

## Conclusion

In conclusion, self-reported HT diagnosis in the SUN Study participants showed enough validity as to be used in this large cohort study. However, our results do not support the use of self-reported BP levels (i.e. a continuous variable) as a valid measurement of usual BP levels.

## List of abbreviations

BP: blood pressure

CI: confidence interval

HT: hypertension

SUN: Seguimiento Universidad de Navarra, University of Navarra Follow-up Study

## Competing interests

The author(s) declare that they have no competing interests.

## Authors' contributions

AA participated in the study design, the acquisition of data, the study analysis, and the first drafting of the manuscript. JJB participated in the acquisition of data and in the interpretation of results. MDR participated in the study design and in the interpretation of data, and provided statistical expertise. MAM have made substantial contributions to conception and design of the study, participated in the statistical analysis and the interpretation of data. All authors participated have revised the manuscript for important intellectual content and read and approved the final manuscript.

## Pre-publication history

The pre-publication history for this paper can be accessed here:


